# Investigating causal associations between inflammatory bowel disease and IgA vasculitis: Univariable and multivariable Mendelian randomization study

**DOI:** 10.1097/MD.0000000000049953

**Published:** 2026-07-24

**Authors:** Bingqing Yi, Yaoyue Luo, Hong Li, Yiran Nie, Shilin Zhu

**Affiliations:** a Department of Nursing, The Second Affiliated Hospital of Hunan University of Chinese Medicine, Changsha, Hunan, China; b College of Nursing, Hunan University of Chinese Medicine, Changsha, Hunan, China.

**Keywords:** causal effect, Crohn disease (CD), IgA vasculitis (IgAV), inflammatory bowel disease (IBD), multivariable Mendelian randomization, ulcerative colitis (UC)

## Abstract

Numerous observational studies have suggested a potential shared genetic background between inflammatory bowel disease (IBD) and IgA vasculitis (IgAV). However, the causal relationship between these 2 conditions remains poorly understood. We performed large-scale two-sample and multivariable Mendelian randomization (MR) analyses (MVMR) to examine whether there is a causal relationship between IBD and IgAV. We employed 4 distinct approaches, including MR-Egger, weighted median, random-effects inverse-variance weighted (IVW), and weighted mode, to conduct the MR analysis. The univariable MR analysis demonstrated that IBD and Crohn disease (CD) were associated with an increased risk of IgAV in the International Inflammatory Bowel Disease Genetics Consortium, with an odds ratio (OR) of 1.17 (95% confidence interval [CI]: 1.06–1.28, *P*_IVW_ = .001) for IBD and an OR of 1.12 (95% CI: 1.01–1.24, *P*_IVW_ = .016) for CD. Ulcerative colitis was associated with an increased risk of IgAV, which was considered suggestive after Bonferroni correction, with an OR of 1.12 (95% CI: 1.01–1.24, *P*_IVW_ = .028). Consistent results were observed after adjusting for potential confounders (drug allergy and upper respiratory tract infection) in MVMR. IBD, ulcerative colitis, and CD were associated with an increased risk of IgAV, with an OR of 1.20 (95% CI: 1.08–1.34, *P*_IVW_ = .001), 1.20 (95% CI: 1.07–1.35, *P*_IVW_ = .002), and 1.13 (95% CI: 1.03–1.25, *P*_IVW_ = .0012). Our study identifies a causal relationship between IBD and IgAV, particularly between CD and IgAV, suggesting that IBD is a potential precursor to IgAV rather than the converse. Further studies on the common mechanisms of these 2 diseases are needed.

## 1. Introduction

Inflammatory bowel disease (IBD) is a chronic, nonspecific intestinal inflammatory disease of unknown etiology, with possible genetic and immune-mediated contributions.^[1]^ The prevalence of IBD has been steadily increasing worldwide since 2000, affecting a substantial proportion of individuals in Western countries.^[2]^ IBD comprises 2 main disorders, Crohn disease (CD) and ulcerative colitis (UC), which differ in various aspects.^[2]^ IBD has moderate genetic susceptibility. Existing genome-wide association studies (GWAS) have reported hundreds of mutations associated with the risk of IBD (UC/CD) and have demonstrated associations with a variety of diseases (multiple sclerosis, type 2 diabetes, rheumatoid arthritis, etc) that share several common genetic loci.^[3,4]^ Immunoglobulin A (IgA) vasculitis (IgAV), also known as Henoch–Schönlein purpura, is a form of vasculitis that involves inflammation of the blood vessels and is characterized by IgA1-dominant immune deposition on diseased vessel walls.^[5]^ The primary clinical presentations of IgAV include purpura, abdominal pain, arthralgia, and renal impairment, whereas a low platelet count is notably absent. The complete elucidation of the pathogenesis underlying IgAV remains a subject of ongoing investigation. It is hypothesized that the development of this condition may be influenced by genetic susceptibility^[6]^ and triggered by environmental factors or precursor infection pathogens. Upon activation of the adaptive immune system, specific antibodies bind to abnormally glycosylated IgA1. This interaction subsequently leads to the deposition of IgA1 immune complexes and the infiltration of inflammatory cells along the walls of small blood vessels, predominantly affecting organs such as the skin, digestive tract, and kidneys.^[7,8]^ In summary, both disease types demonstrate inherent genetic susceptibility, with genetic factors exerting a significant influence on disease pathogenesis and development.

In recent years, an increasing number of case reports and series have described the occurrence of IgAV in IBD patients, which multiple studies consider an adverse event caused by anti-tumor necrosis factor‑alpha (anti-TNF-α) therapy. However, some IBD patients still develop IgAV without using biologics and after completely excluding drug-induced factors. This indicates that anti-TNF-α therapy is not the only cause of IgAV in patients with IBD, and other factors should be considered. A case report study reported the occurrence of IgAV in 2 children from a family with a documented history of IBD, indicating a plausible shared genetic mechanism between these conditions.^[9]^ However, limited research has explored the genetic aspects of IgAV and IBD, with some studies merely establishing a certain association based on observational data,^[10,11]^ thereby necessitating cautious exclusion of potential confounding factors. Mendelian randomization (MR), which leverages genetic variants as instrumental variables (IVs), is a method for inferring causality between an exposure and an outcome. Therefore, our study aimed to explore the causal association between IBD (including UC and CD) and IgAV using univariable and multivariable MR (MVMR) analyses.

## 2. Materials and methods

### 2.1. Study design

**Figure 1.**
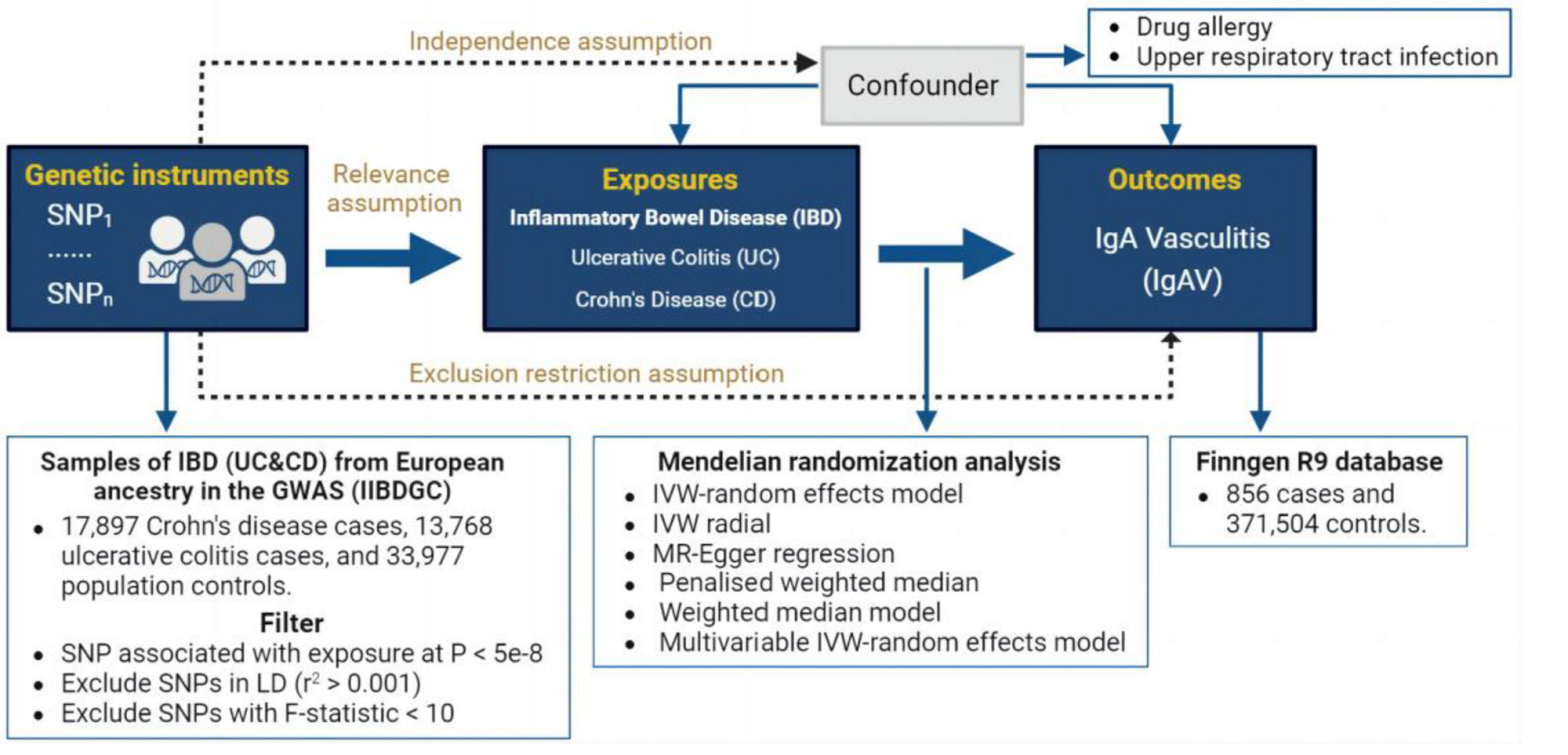
Mendelian randomization concept and assumptions. Schematic illustration depicting the causal relationship between IBD and IgAV. CD = Crohn disease, IBD = inflammatory bowel disease, IgAV = IgA vasculitis, IVW = inverse-variance weighted, LD = linkage disequilibrium, MR = Mendelian randomization, SNPs = single-nucleotide polymorphisms, UC = ulcerative colitis.

To investigate the causal link between IBD (including UC and CD) and IgAV, we began with a two-sample univariable MR (UVMR) study. Following that, reverse MR was conducted to investigate the presence of reverse causality between the variables of interest. We proceeded to execute MVMR. MVMR was conducted to account for the impact of additional exposure-related confounding factors associated with IgAV. Notably, we selected the 2 most prevalent exposure factors identified in the IgAV guidelines,^[12]^ namely drug allergy and upper respiratory tract infection, as essential components of our analysis. Figure 1 illustrates the study design and data sources in a schematic diagram. All utilized data consist of publicly available GWAS summary statistics, thereby obviating the need for additional ethical approval or informed consent.

### 2.2. Data sources

The GWAS data utilized for IBD analysis were sourced from the International Inflammatory Bowel Disease Genetics Consortium.^[13]^ Diagnosis of IBD was established through radiological, endoscopic, and histopathological evaluations, in accordance with accepted clinical criteria for IBD inclusion. Specifically, we focused on individuals of European ancestry, including 17,897 CD cases, 13,768 UC cases, and 33,977 population controls. Additionally, we employed a large-scale GWAS meta-analysis study,^[14]^ as a validation dataset with the following sample sizes (cases/controls: IBD: 25,042/134,915, CD: 12,194/28,072, UC: 12,366/133,609). Diagnosis of IBD was based on accepted endoscopic, histopathological, and radiological criteria. For IgAV, GWAS data were extracted from the most recent version of the FinnGen R9 database, a research initiative that integrates genomic data with national healthcare register data. The updated R9 database offers expanded coverage of IgAV patients, encompassing 856 cases and 371,504 controls. Diagnosis of IgAV was based on coded discharge or death records in accordance with the International Classification of Diseases.

The data on the exposure factor for upper respiratory tract infection were obtained from the FinnGen database, which includes a comprehensive cohort of 35,847 cases and 182,945 controls. Additionally, data on personal history of allergy were sourced from the UK Biobank, specifically focusing on cases with a history of allergy to other drugs, medications, and biological substances. This dataset consisted of 2694 cases and 460,136 controls.

### 2.3. IV selection

In our study, single-nucleotide polymorphisms (SNPs) were selected as IVs, limited to those meeting genome-wide significance thresholds (*P* < 5 × 10^−8^) in the context of IBD. For IgAV within the FinnGen database, we set a more lenient threshold of *P* < 1 × 10^−5^ due to the limited number of SNPs available for MR analysis at a lower threshold. To ensure the independence of each SNP, we applied a linkage disequilibrium factor (*r*^2^) of 0.001 and a clumping window width of 10,000.^[15]^ The strength of each SNP as an instrument was evaluated using the *F*-statistic, calculated as the square of the SNP’s exposure association divided by the square of its standard error. An *F*-statistic exceeding 10 suggests that instrumental bias is unlikely to be weak.^[16]^ Detailed results for all selected SNPs are presented in Table S1, Supplemental Digital Content 1.

### 2.4. MR and sensitivity analysis

Before conducting the analysis, we standardized the exposure and outcome data by aligning the effect alleles to the forward strand. Palindromic genetic variants were excluded from further MR analysis.^[17]^ We employed 4 distinct approaches, including MR-Egger, weighted median, random-effects inverse-variance weighted (IVW), and weighted mode, to conduct the MR analysis and estimate the causal relationship between IBD and IgAV. IVW MR, based on inverse-variance weighting, was employed as the primary analysis, incorporating SNP-specific estimates calculated using Wald ratios, assuming the absence of directional pleiotropic effects for each SNP.^[18]^ Cochran’s *Q* test was used to assess IV heterogeneity, with a *P*-value < .05 indicating the absence of heterogeneity.^[19]^ Furthermore, a leave-one-out analysis was performed by sequentially excluding each SNP and employing the IVW approach on the remaining SNPs to evaluate the potential impact of specific variants on the estimates.^[20]^ To assess the extent of horizontal pleiotropy, Mendelian Randomization Pleiotropy RESidual Sum and Outlier (MR-PRESSO) was utilized to aggregate the residuals for each SNP, enabling the identification of outlier SNPs contributing to overall pleiotropy.^[20]^ We applied MR Steiger filtering to determine the direction of causality for each IV on exposures and outcomes. The Steiger filtering method assumes that a valid IV should explain more variation in exposure than in outcome, with the direction of the instrument classified as “TRUE” if it meets the criteria and “FALSE” otherwise.^[21]^

### 2.5. MVMR analysis

To investigate the genetic relationships between variables and their associations with outcomes, MVMR analyses were performed using the IVW method. The IVW method assumes that all variants employed as IVs are “valid,” implying that the effect of a SNP on the outcome is mediated solely through its effect on the exposure or risk factor. These analyses aimed to elucidate the genetic connections among the variables and their implications for the observed outcomes.^[22]^

### 2.6. Statistical analysis

The MR analyses were performed using the R (version 4.3.0; The R Foundation [R Core Team], https://www.r‐project.org/) computational environment, leveraging the “TwoSampleMR” (MRC Integrative Epidemiology Unit [IEU], University of Bristol, https://mrcieu.github.io/TwoSampleMR/) and “MR-PRESSO” (Dr Marie Verbanck, https://github.com/rondolab/MR‐PRESSO) packages. For specific figures, the R package “forestploter” (Alimu Dayimu, https://cran.r-project.org/package=forestploter) was utilized. Given multiple testing, *P*-values below .016 (0.05/3) were considered robustly significant for the MR analysis of the 3 exposures after Bonferroni correction. A *P*-value between .002 and .05 was considered suggestive, while a *P*-value above .05 was considered not significant.

## 3. Results

### 3.1. Association of IBD and IgAV by UVMR

**Figure 2.**
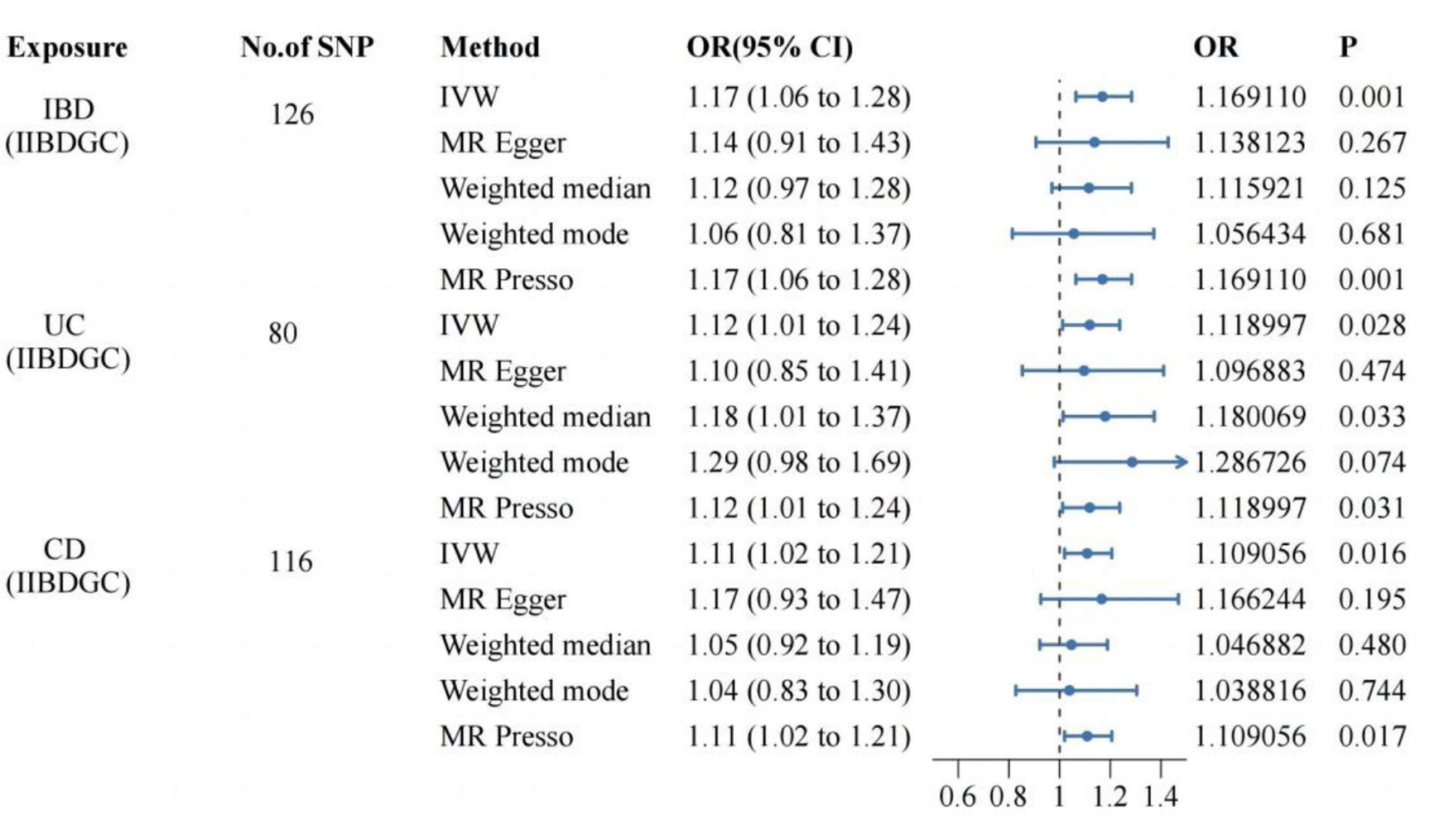
Forest plots for the associations of IgAV with different univariable Mendelian randomization analyses of IBD, UC, and CD from the IIBDGC database. CD = Crohn disease, CI = confidence interval, IBD = inflammatory bowel disease, IgAV = IgA vasculitis, IIBDGC = International Inflammatory Bowel Disease Genetics Consortium, IVW = inverse-variance weighted, OR = odds ratio, SNP = single-nucleotide polymorphism, UC = ulcerative colitis.

**Figure 3.**
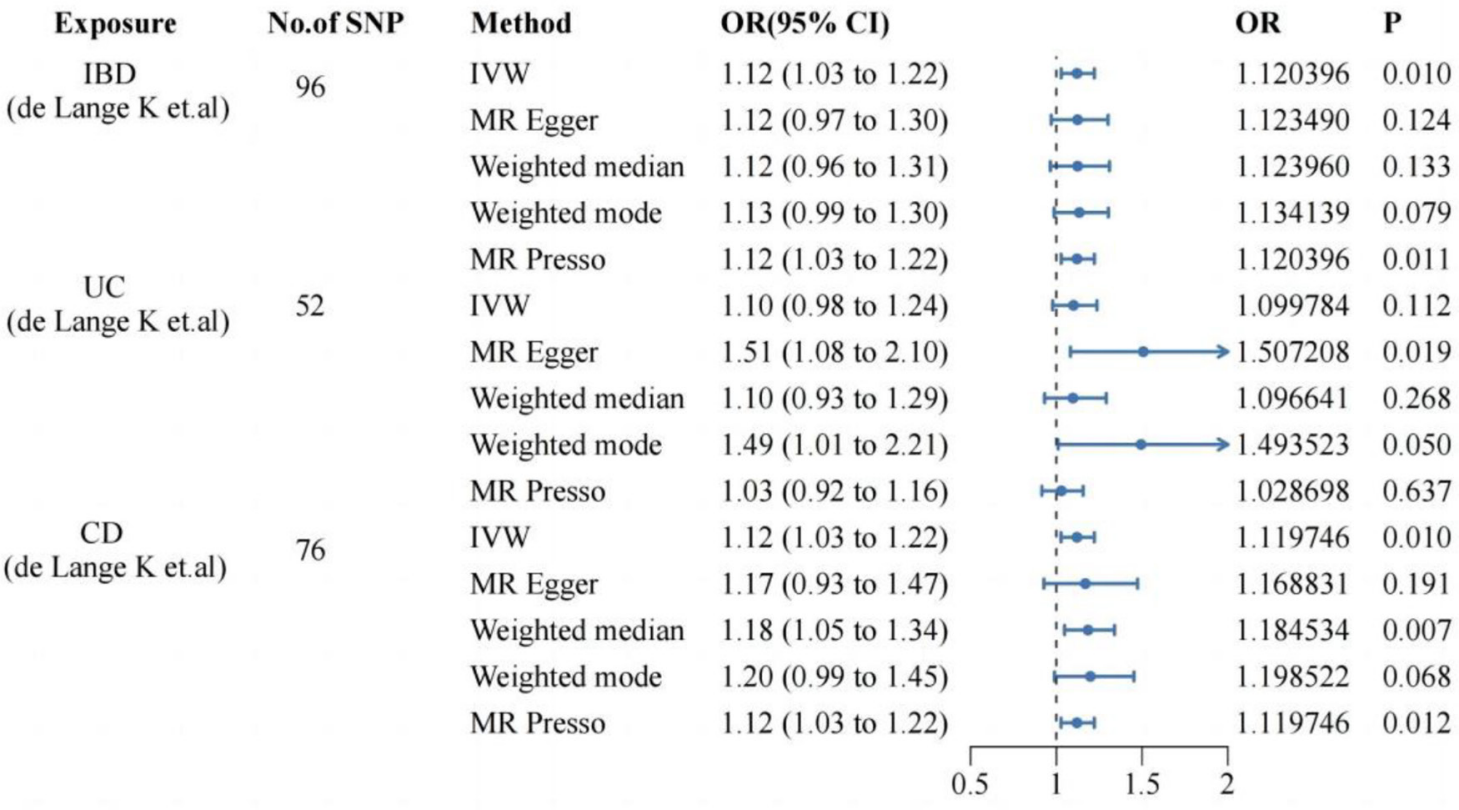
Forest plots for the associations of IgAV with different univariable Mendelian randomization analyses of IBD, UC, and CD from the de Lange et al^[14]^ database. CD = Crohn disease, CI = confidence interval, IBD = inflammatory bowel disease, IgAV = IgA vasculitis, IVW = inverse-variance weighted, OR = odds ratio, SNP = single-nucleotide polymorphism, UC = ulcerative colitis.

For the analysis of IgAV in conjunction with IBD, a total of 128 independent SNPs were identified as genetic IVs for IgAV. Following outcome harmonization, 2 SNPs (rs11768997 and rs140143) were excluded, resulting in a final set of 126 SNPs for analysis. The genetically predicted increase in IBD (per log-odds ratio [OR]) was associated with a higher risk of IgAV, with an OR of 1.17 (95% confidence interval [CI]: 1.06, 1.28) and a *P*-value of .0011 (IVW method). After Bonferroni correction, the *P*-value was .003, which was considered robustly significant. Similarly, for the analysis of IgAV in relation to UC, 83 independent SNPs were identified as genetic IVs. Following outcome harmonization, 3 SNPs (rs10870077, rs140143, and rs1927681) were excluded, leaving 80 SNPs for analysis. A genetically predicted increase in UC (per log-OR) was associated with a higher risk of IgAV, with ORs of 1.12 (95% CI: 1.01, 1.24) and a *P*-value of .028 (IVW method). After applying Bonferroni correction, the resulting *P*-value of .084 was not considered statistically significant. Furthermore, for the analysis of IgAV in relation to CD, 119 independent SNPs were identified as genetic IVs. The mean *F*-statistic for these SNPs was 343.5, ranging from 114.3 to 3044.4. Following outcome harmonization, 3 SNPs (rs11768997, rs140143, and rs1927681) were excluded, resulting in a set of 116 SNPs for analysis. A genetically predicted increase in CD (per log-OR) was associated with a higher risk of IgAV, with ORs of 1.11 (95% CI: 1.02, 1.21) and a *P*-value of .015 (IVW method). After applying the Bonferroni correction, the resulting *P*-value of .045 was considered statistically significant. Additional findings from different detection methods are presented in Figure 2. In the validation dataset (de Lange et al^[14]^), our findings demonstrate consistent outcomes. The genetically predicted increase in the log-OR for IBD showed a strong correlation with elevated susceptibility to IgAV. The ORs, along with their corresponding 95% CIs, for IBD and CD were determined as 1.12 (1.03, 1.22) (*P*_IVW_ = .009) and 1.20 (1.03, 1.22) (*P*_IVW_ = .010), respectively. However, no significant association was observed between UC and IgAV (1.10 [0.98, 1.24], *P*_IVW_ = .11). After applying the Bonferroni correction, the *P*-values for IBD and CD remained robustly significant, whereas UC did not show significance. Additional findings from different detection methods are presented in Figure 3. Detailed information on these genetic variants is provided in Table S1, Supplemental Digital Content 1. Scatter plots of the primary MR analysis and leave-one-out sensitivity analysis are presented in Figures S1 and S2, Supplemental Digital Content 2.

Sensitivity analyses comparing estimates obtained from weighted medians with those from IVW showed no significant differences for each database. MR-Egger intercept analysis did not reveal any evidence of pleiotropic effects. MR-PRESSO identified a potential outlier SNP associated with UC and IgAV in the validation dataset (de Lange et al^[14]^), specifically “rs9271176.” Moreover, MR-PRESSO did not identify any other potential outliers. Cochran’s *Q* statistic *P*-values were all >.05, indicating no significant heterogeneity in individual instrument effects within each database. Steiger filtering did not detect any SNPs with an orientation of “FALSE.” Detailed records of the aforementioned MR and sensitivity analyses are provided in Tables S2 and S3, Supplemental Digital Content 4.

### 3.2. Association of IBD and IgAV by MVMR

**Figure 4.**
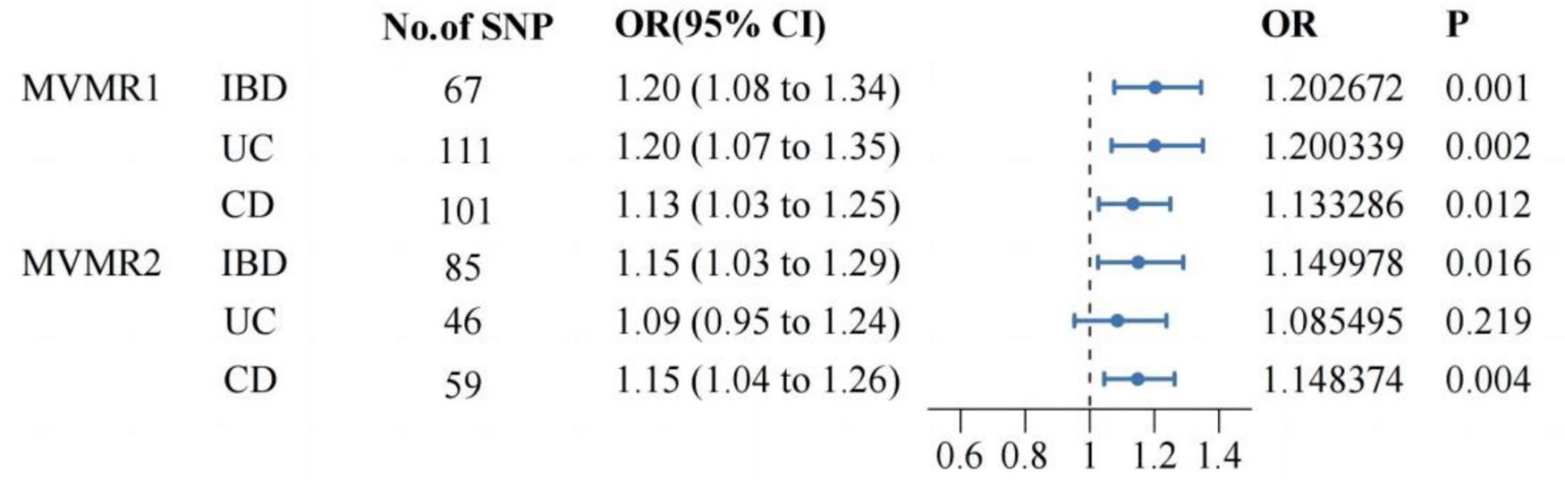
Forest plots for the associations of IgAV with multivariable Mendelian randomization analyses (IVW method) of IBD, UC, and CD. MVMR1: IBD, UC, and CD from the IIBDGC database. MVMR2: IBD, UC, and CD from the de Lange et al^[14]^ database. CD = Crohn disease, CI = confidence interval, IBD = inflammatory bowel disease, IgAV = IgA vasculitis, IIBDGC = International Inflammatory Bowel Disease Genetics Consortium, IVW = inverse-variance weighted, MVMR = multivariable Mendelian randomization, OR = odds ratio, SNP = single-nucleotide polymorphism, UC = ulcerative colitis.

The results of our MVMR analyses are presented in Figure 4. We conducted MVMR analyses on 2 distinct datasets, where the International Inflammatory Bowel Disease Genetics Consortium dataset was referred to as MVMR1, while the validation set (de Lange et al^[14]^) was denoted as MVMR2. In the MVMR1 analysis, after controlling for confounding variables, the *P*-values concerning the causality of IBD and CD in relation to IgAV exhibited minimal alterations compared with UVMR. However, in contrast to the findings of UVMR, we found that the *P*-value associated with UC achieved statistical significance. Specifically, the *P*-values were as follows: .001 for IBD, .002 for UC, and .012 for CD (IVW method). In the MVMR2 analysis, our findings indicate a lack of a significant causal relationship between UC and IgAV, as supported by a *P*-value of .22. We identified a significant causal relationship between both CD and IBD and IgAV, with corresponding *P*-values of .016 and .004, respectively. Notably, even after applying Bonferroni correction, the presence of a causal relationship remains evident.

### 3.3. Reverse MR analysis

In the context of investigating the potential reverse causality of IgAV in relation to IBD, UC, and CD, we identified 2 SNPs associated with the outcome variables within the exposure for IBD (rs7255954 and rs2071538). Following the MR analysis, the resulting *P*-values for IBD were all found to be significantly >.05, indicating the absence of reverse causality. As for UC and CD, MR Steiger identified and excluded 1 SNP (rs2071538), leaving only 1 SNP available for inclusion in the analysis. Consequently, a valid MR analysis could not be performed for UC and CD due to insufficient SNP representation.

## 4. Discussion

Based on the results of this study, there is evidence supporting a causal relationship between IBD and IgAV, but not the reverse. In particular, there is an association between CD and IgAV. Although a causal association between UC and IgAV was identified in the MVMR, no significant causal relationship was found in the UVMR or the validation set. Therefore, we consider this association to be suggestive and to warrant further investigation into this connection.

Several prior epidemiological cohort studies have proposed a potential association between IgAV and IBD.^[11,23,24]^ It has been observed that patients with IBD may develop IgAV, particularly in the context of anti-TNF-α therapy. Nevertheless, some studies also suggest the occurrence of IBD without the administration of anti-TNF-α treatment.^[24]^ Rasmussen et al conducted an investigation encompassing 43 cases of IBD with IgAV. Their findings revealed that, generally, IBD precedes IgAV, and the use of anti-TNF-α therapy appears to contribute to the onset of vasculitis.^[11]^ However, it is important to acknowledge that these studies are limited by sample size, with some comprising only case reports and lacking control groups, which can introduce bias into the results.

Currently, emerging evidence suggests a potential association between genetic factors and the pathogenesis of both IBD and IgAV. Notably, candidate loci implicated in genetic susceptibility for IgAV include human leukocyte antigen (HLA)-DR beta chain 1 and HLA class I.^[25]^ A study conducted in Japan revealed a frequent association between the HLA class region, particularly the HLA-DR beta chain 1 locus, and UC.^[26,27]^ These findings highlight the potential relevance of genetic factors in the pathogenesis of IBD and IgAV. Importantly, our results identified that rs9273363 (HLA-DQ beta chain 1), previously reported to be associated with IBD^[28]^ and IgAV,^[29]^ was one of the crosslinks between these 2 diseases (*P*-value = .0208; Table S1, Supplemental Digital Content 1), providing compelling evidence of a genetic-level causal association between IBD and IgAV.

Both IgAV and IgA nephropathy (IgAN) exhibit immunopathology characterized by IgA deposition, and abnormal IgA1 glycosylation is their shared major pathogenic mechanism. Specifically, IgAV is considered a systemic form of IgAN.^[5]^ Previous investigations have established a genetic causal link between IgA nephropathy and IBD, consistent with conclusions from numerous observational studies. For instance, in autologous kidney biopsies from IBD patients, IgAN has been identified as the most prevalent renal biopsy diagnosis, with a significantly higher prevalence compared with non-IBD kidney biopsy specimens.^[30]^ These results possibly indicate excessive IgA production in individuals with IBD. The intestine is one of the vital immune organs in the human body, and gut-associatedlymphoid tissue serves as a crucial site for antigen presentation and adaptive immune induction within the intestinal wall. Microbial colonization of the gut increases IgA production by the gut-associated lymphoid tissue. Approximately 80% of plasma cells, which produce IgA, are located in the lamina propria of the intestine, making it the major site of IgA production in the human body. The role of IgA in the gut is unquestionably crucial for regulating the gut bacterial community, maintaining a suitable distribution of bacteria, and supporting overall immune homeostasis.^[31]^ Previous research suggests that IBD is associated with immune activation and heightened B-cell stimulation in Peyer’s patches and mucosal lymph nodes,^[11,32]^ which serve as the primary sources of IgA-producing cells.^[33,34]^ This immune dysregulation may lead to the excessive production of IgA, particularly IgA1, and its subsequent deposition in distant tissues. In addition, immunological dysregulation is a hallmark feature of both IgAV and IBD. Wang et al emphasized that IBD is characterized by T cell–mediated intestinal inflammation, which can result in profound dysregulation of polymeric immunoglobulin A production and impaired transport and clearance mechanisms.^[35]^ Consequently, deposition of mesangial-dominantIgA may occur. Extensive infiltration of T helper 17 (Th17) cells was observed in the inflamed intestines of IBD patients.^[36]^ Interestingly, in IgAV patients, there is a notable increase in the proportion of circulating Th17 cells and elevated serum interleukin-17A levels.^[37]^ These findings suggest that dysregulation of the Th17/regulatory T cells axis may be a common factor linking IBD and IgAV. These shared immunological abnormalities may constitute a potential mechanism underlying the immune responses observed in IgAV and IBD.

In our study, we employed MR to establish a causal link between IBD and IgAV. Using genetic evidence, our findings not only reinforce subsequent observational clinical studies but also lay the foundation for mechanistic investigations in this realm. There were some limitations to our research. First, we extracted publicly available pooled data for the GWAS analysis. However, it was challenging to ascertain whether there was any overlap in subjects between the MR analyses conducted on the 2 samples. Second, the source of our research data may introduce bias due to sample selection and misclassification.^[38]^ Nevertheless, by conducting multivariable analyses, we aimed to mitigate this bias. Furthermore, as the sample size increases, the impact of such biases is expected to diminish gradually. Third, our study population consisted of individuals of European descent, which limits the generalizability of our findings to other ethnic populations. Moreover, we emphasize the importance of conducting further observational studies and laboratory-based investigations to validate and expand upon our current findings. By consolidating evidence from multiple research approaches, we can advance the knowledge base and provide a more robust understanding of the intricate relationship between IBD and IgAV.

## Acknowledgments

This study was conducted using resources from the International Inflammatory Bowel Disease Genetics Consortium, IEU Open GWAS, and FinnGen database. We would like to thank all participants.

## Author contributions

**Data curation:** Bingqing Yi.

**Resources:** Bingqing Yi.

**Methodology:** Hong Li.

**Formal analysis:** Hong Li.

**Software:** Bingqing Yi.

**Supervision:** Hong Li.

**Validation:** Yiran Nie.

**Visualization:** Yiran Nie.

**Writing – original draft:** Bingqing Yi.

**Writing – review & editing:** Yaoyue Luo, Shilin Zhu.










