# Supplementary material for: Investigating causal associations between inflammatory bowel disease and IgA vasculitis: Univariable and multivariable Mendelian randomization study
Source: Medicine (Baltimore). 2026 Jul 24;105(30):e49953. doi: 10.1097/MD.0000000000049953 (PMC13406238; doi:10.1097/MD.0000000000049953)
Supplement: Supplementary file 1 [file medi-105-e49953-s001.doc]

**Table S1. Data sources used to identify genetic variants in MR using IIBDGC database**

| Traits | SNP | P-value | EA | OA | Beta | SE | F | R2 |
| --- | --- | --- | --- | --- | --- | --- | --- | --- |
| **Crohn's**  **disease** | rs10758669 | 0.318900685 | A | C | 0.336192321 | 0.337298831 | 534.4416384 | 0.010198014 |
|  | rs10798069 | 0.849560242 | T | G | -0.131097362 | 0.691151775 | 128.7855366 | 0.002476608 |
|  | rs10800309 | 0.083543262 | G | A | 0.961707183 | 0.555743591 | 191.5831955 | 0.003679793 |
|  | rs10878302 | 0.974085461 | A | T | -0.021020922 | 0.647100848 | 169.7129657 | 0.003261095 |
|  | rs10956252 | 0.995848017 | G | C | 0.002376017 | 0.456595949 | 346.7251054 | 0.006639862 |
|  | rs10995271 | 0.012209556 | C | G | 0.665057313 | 0.265382914 | 884.0730782 | 0.01675775 |
|  | rs11117431 | 0.831036646 | G | A | 0.078955584 | 0.370036846 | 369.4016465 | 0.007071052 |
|  | rs11152949 | 0.501597275 | G | A | -0.282839618 | 0.420905647 | 407.2759956 | 0.007790391 |
|  | rs11159833 | 0.838843406 | T | C | 0.116026851 | 0.570512218 | 198.9256222 | 0.003820282 |
|  | rs11167518 | 0.230035928 | A | C | -0.375087139 | 0.312503262 | 614.3871773 | 0.011705648 |

| rs11185982  rs11236797  rs11691685  rs11713774  rs11793497  rs12411259  rs1250573  rs1267501  rs12694846  rs12796489  rs1292053  rs12949918  rs1297258  rs13001325  rs13407913  rs1363907  rs1456896  rs1517352 | 0.985947017  0.099698457  0.540952119  0.00277245  0.046030799  0.291216667  0.507792391  0.259354952  0.793411446  0.911112454  0.101621766  0.719308161  0.902468676  0.959849065  0.813283362  0.482561127  0.65526418  0.123622368 | C  A  G  C  G  A  A  C  G  A  G  C  T  T  G  A  T  C | T  C  A  T  A  G  G  T  A  C  A  T  C  C  A  G  C  A | 0.011937346  0.448923574  -0.350733415  1.593063575  0.580006749  0.423140871  0.23546329  -0.996001317  0.133222093  0.052568928  0.87812084  -0.169121783  0.047502397  0.021127503  -0.101545744  0.342705338  -0.244299091  0.963419435 | 0.677730234  0.272683513  0.573680773  0.532458428  0.290714057  0.400907981  0.355534854  0.883044826  0.508707938  0.470898114  0.536407144  0.470588518  0.387637332  0.419671196  0.42992804  0.488052644  0.547190049  0.625700384 | 159.5112889  850.4643698  191.0808125  224.3298168  732.729022  343.9917124  453.7483417  121.3346997  266.556656  1494.620023  213.626424  275.4558472  413.7439577  368.8688751  335.6816108  267.4832891  212.9492466  159.22382 | 0.003065667  0.016130968  0.003670179  0.004306058  0.013928957  0.006587861  0.008671607  0.002333659  0.005112467  0.028006646  0.004101447  0.005282249  0.007913131  0.007060925  0.006429736  0.005130148  0.004088499  0.003060159 |
| --- | --- | --- | --- | --- | --- | --- | --- |

| rs1569328  rs1646019  rs17129991  rs17293632  rs17388425  rs17391694  rs17622378  rs17694108  rs181826  rs1847472  rs2024092  rs212388  rs2153283  rs2227551  rs2270395  rs2284553  rs2395022  rs2413583 | 0.023131429  0.974742448  0.72474504  0.356348656  0.028611436  0.835360859  0.408316208  0.732348456  0.877088179  0.731038554  0.266903104  0.430144206  0.477392667  0.178153038  0.613009868  0.221630538  0.939118583  0.808803916 | T  T  T  T  G  T  G  A  A  A  A  T  A  T  T  G  C  T | C  C  C  C  C  C  A  G  C  C  G  C  C  G  C  A  A  C | 1.316018862  -0.015030751  0.224694774  -0.395863192  0.950799031  -0.125202644  0.226381594  -0.218458465  0.079119392  0.224909645  -0.435148716  -0.39449095  -0.373040513  -0.684152944  -0.214567113  0.595370765  0.050625169  -0.087095486 | 0.579423156  0.474741199  0.638112079  0.429192111  0.434392985  0.602425011  0.27378455  0.638761394  0.511564861  0.654293357  0.391947259  0.500023443  0.525037682  0.508110967  0.424228653  0.487128847  0.662826273  0.359944298 | 175.3582704  273.2288845  184.9688628  310.5204774  346.7478528  158.6512335  929.7362745  132.7364901  241.8920172  170.793284  386.184214  261.3176849  209.5196998  203.5791507  292.8051198  268.5368252  128.7812083  641.8568279 | 0.003369206  0.005239768  0.003553201  0.005950661  0.006640294  0.003049188  0.017608063  0.002552392  0.004641603  0.003281785  0.007389928  0.005012489  0.004022918  0.003909302  0.005613078  0.00515025  0.002476524  0.012222618 |
| --- | --- | --- | --- | --- | --- | --- | --- |

| rs2538470  rs259964  rs2641348  rs26528  rs2847293  rs28999107  rs2974935  rs3024505  rs303429  rs3129871  rs3184504  rs3197999  rs34592089  rs34779708  rs34787213  rs34804116  rs35164067  rs35320439 | 0.630513289  0.997335127  0.941821735  0.092643951  0.944227485  0.27016904  0.94372254  0.160521577  0.111608569  0.038924591  0.178300952  0.21075752  0.657943709  0.264887622  0.237525171  0.492669824  0.785089595  0.093766792 | G  G  G  C  T  T  T  A  T  C  C  A  A  G  T  A  A  C | A  A  A  T  A  G  G  G  C  A  T  G  G  T  C  C  G  T | 0.321219567  0.002304701  -0.041094646  0.68063749  0.02831533  0.632910107  0.04562102  0.565363466  - 1.026801494  - 1.187063534  0.980983282  -0.402139072  0.615422806  0.414904393  -0.638489949  0.367745872  -0.12135409  - 1.146392562 | 0.667809878  0.690044887  0.563091745  0.404752806  0.404750436  0.57397787  0.646264032  0.402875792  0.645379387  0.574853848  0.728811104  0.321329474  1.389988265  0.372140536  0.540545649  0.536019065  0.445021727  0.684064695 | 135.0311602  131.4043259  147.1972123  372.4912161  390.5797181  188.1256182  148.4479146  378.5524883  145.3010372  180.5627359  118.9463253  509.4871078  207.7634518  430.9114587  281.7208742  217.8920183  348.490172  157.3728551 | 0.002596402  0.002526841  0.002829671  0.00712977  0.007473411  0.003613622  0.002853645  0.007244947  0.002793321  0.003468854  0.002287828  0.009726473  0.003989332  0.008238766  0.005401741  0.004183  0.006673437  0.003024693 |
| --- | --- | --- | --- | --- | --- | --- | --- |

| rs35730213  rs36016881  rs3776414  rs3801810  rs3853824  rs438475  rs4703855  rs4795397  rs516246  rs559928  rs56163845  rs6062496  rs6074022  rs6111031  rs61839660  rs640466  rs6456426  rs6500315 | 0.860127753  0.068859499  0.939858017  0.057388096  0.393516424  0.39669618  0.443873524  0.14021368  0.851167805  0.963501855  0.687592746  0.726712829  0.503394758  0.950905273  0.928869478  0.127405481  0.863764219  0.079991176 | C  G  G  A  C  A  T  G  T  C  G  A  T  T  T  C  A  G | G  A  T  G  T  G  C  A  C  T  A  G  C  C  C  T  C  A | 0.069141326  0.917561329  -0.045125305  1.126105121  -0.51252071  -0.460672257  0.527545352  -0.54069655  0.081943077  -0.028082301  0.215671106  -0.14798964  -0.378418643  -0.016011347  0.073984031  1.009324726  -0.083816615  -0.821099499 | 0.392376824  0.504337332  0.598095577  0.592579868  0.600663461  0.543542709  0.688999604  0.366574982  0.436730205  0.613691941  0.536329808  0.423430603  0.565514721  0.260051139  0.828792041  0.662106974  0.488486028  0.469002123 | 482.7207712  178.7607455  192.7228374  205.9554656  158.9996101  302.9370237  117.1499566  456.7157155  341.057448  155.5645618  187.9933425  369.3711252  181.2462553  1130.753027  186.8635829  139.9510422  256.3796865  385.6662509 | 0.009220196  0.003434354  0.003701601  0.003954753  0.003055863  0.005806179  0.002253354  0.008727822  0.006532034  0.002990041  0.00361109  0.007070471  0.00348194  0.021333855  0.003589467  0.002690748  0.004918236  0.007380089 |
| --- | --- | --- | --- | --- | --- | --- | --- |

| rs6561151  rs6651252  rs6679677  rs6702421  rs6738394  rs6738490  rs6740462  rs6827756  rs6908425  rs7015630  rs7085798  rs71624119  rs7194886  rs7236492  rs72727394  rs727563  rs7438704  rs7517847 | 0.628295566  0.368470923  0.279182531  0.467000969  0.13372083  0.923601695  0.85585132  0.329735746  0.964905413  0.664947586  0.825240174  0.224406178  0.868230122  0.463673499  0.247967365  0.761750224  0.28613576  0.037801759 | A  C  A  T  A  C  A  C  C  C  A  A  T  T  T  T  G  G | G  T  C  C  G  T  C  T  T  T  C  G  C  C  C  C  A  T | -0.199111099  0.457671997  -0.399160149  0.429474382  -0.965109608  -0.020705108  -0.091466357  0.598795661  -0.023269512  0.280733011  -0.061362906  0.85356269  0.035395267  0.448050443  -0.673638275  -0.180500015  -0.621136113  0.301658061 | 0.411278441  0.508896926  0.368855859  0.590450478  0.643582632  0.215907925  0.503509229  0.614372281  0.528867754  0.648205693  0.2778988  0.702581505  0.213343995  0.611413079  0.583084866  0.595349589  0.58233127  0.145237542 | 392.7959212  261.9114501  314.991982  220.9493901  155.7625559  1357.254132  200.411031  152.0417305  190.4818825  144.0689408  799.5424725  162.8036001  1348.352663  134.3804793  177.9691615  142.6883107  168.0813934  3044.48544 | 0.007515497  0.005023821  0.006035833  0.004241445  0.002993835  0.025498274  0.003848699  0.002922528  0.003658717  0.002769701  0.015179781  0.003128744  0.025335282  0.002583923  0.003419198  0.002743231  0.003229845  0.055438461 |
| --- | --- | --- | --- | --- | --- | --- | --- |

|  | rs7608910  rs76906269  rs7773324  rs7786444  rs77981966  rs780094  rs7848647  rs7969592  rs915286  rs9264942  rs9457247  rs9491892  rs9494844  rs9554587  rs9594766  rs9889296 | 0.297725533  0.672278271  0.350298544  0.902636668  0.213467751  0.033924248  0.004964094  0.567353416  0.302198931  0.182837653  0.72101426  0.73498303  0.32283293  0.304532691  0.211909381  0.739260105 | G  G  A  T  T  C  C  G  A  C  T  G  A  G  A  A | A  A  G  C  C  T  T  A  G  T  C  T  C  A  G  G | -0.433205648  -0.321541508  -0.58012928  -0.102788063  -0.92631504  0.924246457  1.049034733  0.390186884  0.752011404  0.480536361  -0.140237581  0.167461141  -0.572868322  -0.778496597  0.847213026  0.119239397 | 0.416014559  0.760103565  0.62111653  0.840243565  0.744574501  0.435765734  0.373407733  0.682200252  0.728884387  0.360744459  0.392708409  0.494708745  0.579443245  0.758205727  0.67867587  0.358255589 | 361.8224478  299.7810014  154.8214085  134.2967452  234.4222645  338.5537874  458.7588048  138.9452375  114.3503935  544.362318  397.1314946  252.0905597  155.1247006  163.9048324  140.2142183  424.3876736 | 0.006926976  0.005746037  0.0029758  0.002582317  0.004498913  0.006484394  0.008766523  0.002671461  0.002199623  0.010385351  0.007597821  0.004836354  0.002981612  0.003149841  0.002695794  0.008115048 |
| --- | --- | --- | --- | --- | --- | --- | --- | --- |
| **Ulcerative**  **colitis** | rs10185424 | 0.513507121 | G | T | 0.359411798 | 0.55007531 | 222.3898631 | 0.004636465 |
|  | rs10460566 | 0.096221137 | A | G | 1.29271726 | 0.77712728 | 116.3627815 | 0.002431348 |

| rs10748783  rs10758669  rs10761659  rs1077773  rs10910092  rs11083840  rs11150589  rs111830527  rs11229555  rs11230563  rs11641184  rs11676348  rs1182188  rs12132349  rs12318183  rs12718244  rs12720356  rs12796489 | 0.82137117  0.318900685  0.039174612  0.844965253  0.392256139  0.52076479  0.931913182  0.020320011  0.97479836  0.378520618  0.826616917  0.101055798  0.450466331  0.85847843  0.247316497  0.464665328  0.893411985  0.911112454 | A  A  G  A  G  G  C  A  T  T  A  T  C  A  A  A  C  A | C  C  A  G  A  T  T  G  G  C  C  C  T  T  C  G  A  C | -0.066403045  0.351525646  0.852474108  0.131663483  0.48092312  -0.474897153  0.052696047  -2.675011959  0.020775101  0.66240645  -0.136636283  - 1.086874397  0.354791618  0.063300428  -0.368908853  -0.543474021  0.07709471  0.061527147 | 0.29410279  0.35268262  0.41335123  0.67331085  0.56213274  0.73952288  0.61677526  1.15283064  0.657630828  0.752200046  0.623788822  0.662826689  0.470148214  0.354999341  0.318879256  0.743277019  0.575384756  0.551143402 | 655.4003022  449.5281272  328.6942258  124.2319733  177.9091616  110.0008864  152.2599619  176.4655278  122.1657291  122.3973444  145.4330204  132.3345855  232.8650503  543.6625743  602.383619  119.1236024  176.4691088  996.4755044 | 0.013541776  0.009327756  0.006837584  0.002595345  0.003712558  0.002298725  0.003179019  0.003682544  0.002552289  0.002557115  0.003036913  0.00276415  0.004853796  0.011259063  0.012460003  0.002488891  0.003682618  0.020444937 |
| --- | --- | --- | --- | --- | --- | --- | --- |

| rs1297256  rs13136827  rs13255292  rs13430791  rs1517352  rs16841904  rs17694108  rs17780256  rs1801274  rs1990760  rs2274351  rs2395022  rs2497318  rs2516440  rs272882  rs2836883  rs3024493  rs34659678 | 0.906170787  0.458350266  0.679607913  0.199696967  0.123622368  0.837375997  0.732348456  0.383340916  0.393240854  0.233927203  0.619904965  0.939118583  0.469824025  0.048746288  0.727575949  0.401312663  0.140075246  0.725235334 | T  C  T  A  C  T  A  C  G  T  T  C  T  A  T  A  A  T | C  T  C  G  A  C  G  A  A  C  C  A  C  G  G  G  C  C | 0.057519691  0.505457277  -0.280273777  - 1.140311278  0.990987852  0.164716208  -0.181408616  -0.487919992  0.241717185  0.68502405  0.338382709  0.048768597  0.492664217  - 1.002246065  -0.117768895  0.208063522  0.435662327  0.167456072 | 0.487993271  0.681607849  0.678631912  0.889190261  0.643604911  0.802510971  0.53042953  0.559697198  0.283122484  0.575503779  0.682243779  0.63851851  0.681637795  0.508546773  0.338074539  0.247907403  0.295262023  0.476444055 | 239.4978032  162.780166  120.230311  113.4844345  138.4859654  114.5089535  177.3738367  198.6040235  707.4586276  167.1573537  120.2991373  127.7512967  120.9305301  208.4963134  451.0553255  997.6632269  656.6499996  228.5841801 | 0.004991358  0.003397923  0.002511956  0.002371349  0.002892265  0.002392706  0.003701428  0.004142624  0.014601691  0.003488975  0.00251339  0.002668671  0.002526548  0.004348067  0.009359149  0.020468807  0.013567247  0.004764991 |
| --- | --- | --- | --- | --- | --- | --- | --- |

| rs35223180  rs36070529  rs3774937  rs3776414  rs4366152  rs4656958  rs4676410  rs4712520  rs4728142  rs4743820  rs4747886  rs4795397  rs4812833  rs483905  rs4947328  rs4973341  rs4976646  rs55808324 | 0.158469197  0.622065538  0.26961915  0.939858017  0.00775879  0.904638871  0.860413149  0.591847603  0.453739649  0.246925989  0.648782494  0.14021368  0.547485502  0.094076027  0.630480239  0.864021949  0.252470981  0.982906541 | T  A  C  G  C  G  A  C  A  T  T  G  A  A  G  T  C  A | G  G  T  T  T  A  G  T  G  C  C  A  G  G  A  C  T  G | 0.556185329  0.363425805  0.565078166  -0.056877573  1.183000889  -0.07554498  -0.067928701  0.340133048  0.379621542  0.737918115  0.305397987  -0.511642224  0.289147137  - 1.159432516  -0.232084943  -0.134567937  0.734373175  -0.014785668 | 0.394390899  0.737284088  0.511873968  0.75386139  0.444337922  0.630574477  0.38629156  0.634386574  0.506705883  0.637318531  0.670534978  0.346877078  0.480684303  0.69249712  0.482453906  0.785767561  0.641728065  0.69010919 | 280.7557532  128.7784463  207.5789495  111.4988974  302.9629534  141.052383  314.5788236  123.133641  222.1426417  131.1929454  126.1178779  469.9416636  255.9615712  142.154819  126.8105385  115.4989208  133.6209615  130.9207442 | 0.005846185  0.00269007  0.004329019  0.002329956  0.00630569  0.002945707  0.006545873  0.002572458  0.004631335  0.002740369  0.00263464  0.00974721  0.005332648  0.002968662  0.002649071  0.002413342  0.002790944  0.002734699 |
| --- | --- | --- | --- | --- | --- | --- | --- |

| rs56167332  rs59418206  rs6062496  rs6111031  rs61893460  rs6426833  rs6466198  rs661054  rs6920220  rs7240004  rs7404095  rs7547569  rs7608910  rs76546301  rs76904798  rs7738430  rs79045992  rs8096327 | 0.671158601  0.272465524  0.726712829  0.950905273  0.104457212  0.04399566  0.535568064  0.248518183  0.317626413  0.008905252  0.716626143  0.253326173  0.297725533  0.512615459  0.12050264  0.435331583  0.369803748  0.19809908 | A  A  A  T  A  A  T  G  A  G  C  C  G  A  T  C  A  G | C  G  G  C  G  G  A  A  G  A  T  T  A  G  C  T  G  A | -0.158163092  0.746299522  -0.156019421  -0.017331166  0.660770812  0.419859975  -0.232295423  0.459261721  -0.418979011  1.592644979  -0.255883492  0.264193536  -0.41108506  -0.54100619  1.249837513  0.229815145  -0.675254249  -0.698636548 | 0.37253551  0.68006004  0.446405556  0.281487217  0.406974133  0.208457014  0.374955011  0.397988915  0.4192526  0.608886002  0.704968468  0.23128055  0.394771792  0.826253019  0.804961577  0.294596754  0.752925237  0.542846939 | 430.5269344  117.9315333  305.657416  885.4414472  348.4312136  1317.867188  409.3677798  336.776648  342.7783802  153.0290028  120.140995  1507.758472  370.1109822  120.213997  123.7439718  332.6454159  123.6684733  199.5638192 | 0.008937003  0.002464046  0.006361414  0.018208304  0.007245183  0.026861881  0.008501509  0.007004539  0.007128477  0.003195025  0.002510094  0.030613914  0.007692518  0.002511616  0.002585176  0.006919209  0.002583603  0.00416256 |
| --- | --- | --- | --- | --- | --- | --- | --- |

|  | rs913678  rs941823  rs9611131  rs9836291  rs9891119  rs9941524 | 0.414109954  0.633117514  0.930179663  0.242333821  0.778408095  0.953382135 | C  C  C  A  C  G | T  T  T  G  A  A | 0.549412009  -0.239257746  0.046370186  -0.339845645  0.156535082  -0.029077138 | 0.672736441  0.501227581  0.529226467  0.290671749  0.55628404  0.497384246 | 121.3439137  211.8506062  246.0188868  574.1615708  175.6496718  227.2364411 | 0.002535163  0.00441771  0.005126566  0.011883181  0.003665581  0.00473703 |
| --- | --- | --- | --- | --- | --- | --- | --- | --- |
| **Inflammatory bowel disease** | rs10142466 | 0.242326878 | G | A | 0.977803791 | 0.836308344 | 110.5944002 | 0.001682029 |
|  | rs10758669 | 0.318900685 | A | C | 0.338425135 | 0.339538995 | 667.3096053 | 0.010063892 |
|  | rs10761659 | 0.039174612 | G | A | 0.650200571 | 0.31527199 | 780.681113 | 0.011753585 |
|  | rs10800309 | 0.083543262 | G | A | 0.658132934 | 0.380316552 | 519.8479411 | 0.007857454 |
|  | rs10878302 | 0.974085461 | A | T | -0.029311388 | 0.902311681 | 110.2787455 | 0.001677236 |
|  | rs10956252 | 0.995848017 | G | C | 0.003376793 | 0.648913805 | 216.4949239 | 0.003287374 |
|  | rs11152949 | 0.501597275 | G | A | -0.360354855 | 0.536259364 | 316.5460382 | 0.004799312 |
|  | rs11185982 | 0.985947017 | C | T | 0.016728256 | 0.949729077 | 102.6328354 | 0.001561131 |
|  | rs11230563 | 0.378520618 | T | C | 0.612417174 | 0.695434392 | 196.9583823 | 0.002991608 |
|  | rs11236797 | 0.099698457 | A | C | 0.537816179 | 0.326678333 | 746.1306422 | 0.011239255 |
|  | rs11641016 | 0 835159484 | G | C | 0 103276184 | 0 496307778 | 258 5131472 | 0 003922898 |

| rs11677953  rs11691685  rs11713774  rs11793497  rs1182188  rs12318183  rs12411259  rs1250566  rs12585310  rs1267499  rs12718244  rs12722515  rs12796489  rs1292053  rs1297258  rs13107612  rs13204742  rs13407913 | 0.192980796  0.540952119  0.00277245  0.046030799  0.450466331  0.247316497  0.291216667  0.675271698  0.341006549  0.244158084  0.464665328  0.521260113  0.911112454  0.101621766  0.902468676  0.021310622  0.812421485  0.813283362 | A  G  C  G  C  A  A  A  A  C  A  A  A  G  T  T  T  G | G  A  T  A  T  C  G  G  G  T  G  C  C  A  C  C  G  A | -0.865699678  -0.451600023  2.241472205  0.627222386  0.579351712  -0.546228009  0.849780436  0.205478026  -0.701903084  - 1.09157787  -0.512118145  0.451799859  0.054740014  1.142194042  0.052779243  1.649732923  0.187032857  -0.126792662 | 0.664996129  0.738664293  0.749179622  0.314379729  0.767721554  0.472151263  0.805130827  0.490490019  0.737155071  0.937253102  0.700393457  0.704391813  0.490346109  0.697718374  0.430698369  0.71651644  0.788159457  0.536819356 | 196.9901264  145.6346315  143.0848355  791.2505541  119.7025636  375.1908605  107.3938683  290.1763497  141.3487555  136.6139331  184.5060934  175.4034342  1740.3755  159.5102249  423.4619955  147.3966387  122.1614866  271.8264403 | 0.002992089  0.002213776  0.002175101  0.011910818  0.001820303  0.005683402  0.001633432  0.004401268  0.002148766  0.002076938  0.002803  0.002665082  0.025829115  0.002424186  0.006409927  0.0022405  0.001857626  0.004124092 |
| --- | --- | --- | --- | --- | --- | --- | --- |

| rs1363907  rs1388585  rs1420098  rs1517352  rs1569328  rs17293632  rs17651741  rs17694108  rs17780256  rs181826  rs1847472  rs1990760  rs2024092  rs2050392  rs2143178  rs2153283  rs2270395  rs2274351 | 0.482561127  0.963024812  0.762571497  0.123622368  0.023131429  0.356348656  0.349158773  0.732348456  0.383340916  0.877088179  0.731038554  0.233927203  0.266903104  0.155501761  0.774153855  0.477392667  0.613009868  0.619904965 | A  A  C  C  T  T  A  A  C  A  A  T  A  A  C  A  T  T | G  G  T  A  C  C  G  G  A  C  C  C  G  G  T  C  C  C | 0.431311394  0.022647348  0.156742582  0.989744689  1.77504131  -0.474245323  -0.811482528  -0.20273335  -0.67484867  0.096044093  0.285431886  0.873420437  -0.601572842  - 1.013714268  -0.122912092  -0.472156271  -0.34270713  0.397662452 | 0.614238073  0.48853029  0.518833602  0.642797529  0.781523782  0.514173471  0.866762708  0.59278196  0.774124684  0.620995461  0.830360952  0.733779731  0.541848839  0.713700378  0.428346087  0.664538637  0.677579068  0.801762996 | 213.2884184  230.4980008  284.234172  190.8789767  121.7891166  273.2890984  100.2550485  195.1157808  142.4524677  207.4107629  134.0219015  141.177169  254.7946351  150.8264961  571.7606854  165.2502924  144.7459139  119.6758992 | 0.003238842  0.00349926  0.004311528  0.002899536  0.001851974  0.004146191  0.001525018  0.002963704  0.002165509  0.00314987  0.002037611  0.002146164  0.003866688  0.002292516  0.008635334  0.002511202  0.002200296  0.001819898 |
| --- | --- | --- | --- | --- | --- | --- | --- |

| rs2297559  rs2328546  rs2395022  rs2488397  rs2497318  rs2538470  rs259964  rs2688608  rs272882  rs2836883  rs2847278  rs2974935  rs3024493  rs3184504  rs34779708  rs34804116  rs34856868  rs35164067 | 0.909095383  0.475048172  0.939118583  0.838041698  0.469824025  0.630513289  0.997335127  0.107738324  0.727575949  0.401312663  0.762522822  0.94372254  0.140075246  0.178300952  0.264887622  0.492669824  0.044939426  0.785089595 | A  C  C  C  T  G  G  T  T  A  T  T  A  C  G  A  A  A | G  T  A  G  C  A  A  G  G  G  C  G  C  T  T  C  G  G | -0.080039479  0.427995388  0.049375671  0.14280336  0.553842547  0.356424447  0.002437117  - 1.254011826  -0.103404829  0.280609573  0.143770757  0.050151617  0.500608363  1.105490932  0.522940785  0.600985676  - 1.384631929  -0.148025353 | 0.70099601  0.599190352  0.646466815  0.698649934  0.76628259  0.741000209  0.729691187  0.779643607  0.296840179  0.334345923  0.475795002  0.710444143  0.339277988  0.82131274  0.469041705  0.875984762  0.690513379  0.542828824 | 156.9313771  185.2064875  171.3366597  208.5671315  131.4905949  138.715932  148.6638707  126.3874178  806.657249  747.0728894  368.7470809  155.365796  681.4746644  118.4643874  342.2000022  102.9685378  153.5024056  295.7379713 | 0.002385087  0.00281361  0.002603452  0.003167375  0.001999204  0.002108827  0.002259719  0.001921763  0.012139922  0.011253289  0.005586337  0.002361349  0.010275324  0.001801508  0.005186247  0.001566229  0.002333094  0.004485245 |
| --- | --- | --- | --- | --- | --- | --- | --- |

| rs35256947  rs35730213  rs36048684  rs367569  rs3776414  rs3801835  rs3853824  rs4692386  rs4703855  rs4743820  rs4795397  rs4976646  rs516246  rs55808324  rs559928  rs56167332  rs6058869  rs6062496 | 0.73838175  0.860127753  0.596370632  0.646510464  0.939858017  0.540245339  0.393516424  0.236292869  0.443873524  0.246925989  0.14021368  0.252470981  0.851167805  0.982906541  0.963501855  0.671158601  0.987974562  0.726712829 | C  C  A  T  G  T  C  C  T  T  G  C  T  A  C  A  T  A | T  G  T  C  T  C  T  T  C  C  A  T  C  G  T  C  C  G | 0.239045478  0.065451311  -0.531873198  0.260445476  -0.051811259  0.518734304  -0.65123612  - 1.024205177  0.54387703  0.933733423  -0.517226748  0.792344473  0.124255194  -0.013319383  -0.029491355  -0.143461551  0.013373894  -0.144220718 | 0.715711248  0.371436  1.004241262  0.567895333  0.68671192  0.846993308  0.763234996  0.864816335  0.710329561  0.806438549  0.350663207  0.692385973  0.662240156  0.621671517  0.644484468  0.337907671  0.887320679  0.412646897 | 170.7067829  682.4001002  114.7523039  248.6444021  184.8295357  122.0950326  124.4718385  106.6687885  139.456211  112.5361515  632.0942198  157.7493885  187.0027229  221.9513262  178.4434907  720.8469322  97.68731217  492.3450993 | 0.002593906  0.010289135  0.001745156  0.003773706  0.0028079  0.001856617  0.001892691  0.001622421  0.002120057  0.00171151  0.009537864  0.002397489  0.002840821  0.003369948  0.002711147  0.010862534  0.001486017  0.007444846 |
| --- | --- | --- | --- | --- | --- | --- | --- |

| rs6074022  rs6111031  rs62037363  rs62434177  rs6456426  rs6466198  rs648541  rs6500315  rs6561151  rs6584281  rs6588248  rs6651252  rs6708373  rs6740462  rs6745185  rs6933404  rs7011507  rs7015630 | 0.503394758  0.950905273  0.035644211  0.675182891  0.863764219  0.535568064  0.278423384  0.079991176  0.628295566  0.825529503  0.031932761  0.368470923  0.915458447  0.85585132  0.79265296  0.322914275  0.505637238  0.664947586 | T  T  C  A  A  T  G  G  A  G  G  C  G  A  G  C  A  C | C  C  T  G  C  A  A  A  G  A  T  T  A  C  T  T  G  T | -0.490847537  -0.017119326  1.047553083  -0.245409676  -0.129201514  -0.369776017  0.830503719  - 1.559911341  -0.292988  -0.064878917  1.268559413  0.750771615  -0.038627718  -0.114089723  -0.24603053  -0.637079407  0.689329099  0.376740008 | 0.733530213  0.278046582  0.498606145  0.585639709  0.752990729  0.596866561  0.766240734  0.89100253  0.605188  0.294317264  0.591333716  0.834801714  0.363876343  0.628047629  0.935950002  0.644499633  1.035578734  0.869883514 | 136.126078  1248.249967  306.3173817  132.1325038  136.1453584  220.9672665  124.3294522  134.4964745  228.6270404  900.54911  220.5704085  122.7742508  594.4034106  162.7750567  123.7234336  200.8523962  101.7272276  101.1051594 | 0.002069536  0.018661723  0.004644951  0.002008944  0.002069829  0.003355057  0.00189053  0.002044812  0.003470955  0.013533839  0.003349051  0.001866926  0.00897424  0.002473681  0.001881333  0.003050574  0.001547377  0.001537929 |
| --- | --- | --- | --- | --- | --- | --- | --- |

| rs71593329  rs7194886  rs7240004  rs7253253  rs72634258  rs72924296  rs744166  rs7523442  rs7547569  rs7608910  rs7657746  rs769177  rs7773324  rs780094  rs7848647  rs78487399  rs913678  rs9264942 | 0.622086425  0.868230122  0.008905252  0.455405662  0.171855889  0.882178006  0.707067231  0.02261284  0.253326173  0.297725533  0.322381214  0.439927324  0.350298544  0.033924248  0.004964094  0.240110685  0.414109954  0.182837653 | G  T  G  T  C  G  G  T  C  G  G  T  A  C  C  C  C  C | T  C  A  G  T  A  A  C  T  A  A  C  G  T  T  G  T  T | 0.340738946  0.063750575  1.971978984  -0.573479438  0.600472899  -0.134677126  -0.184207685  0.887856621  0.202350124  -0.413214546  0.795085327  0.311808986  -0.73897085  1.374858726  1.119699373  -0.945856328  0.601788784  0.76523888 | 0.691300578  0.384254836  0.753909638  0.768292864  0.439499673  0.908698731  0.49017767  0.389425633  0.177141533  0.396816773  0.803460282  0.403732422  0.791180562  0.648221389  0.39856107  0.805182712  0.736870033  0.574474084 | 201.3900585  516.6799543  137.0825694  103.5755063  307.5043222  105.5241134  321.5582334  510.3779514  3614.114102  503.5799317  183.1901817  222.6473368  120.6047674  192.9176305  509.0115115  200.7721083  138.9956608  269.9170993 | 0.003058715  0.007809944  0.002084048  0.001575447  0.004662865  0.001605039  0.004874934  0.00771542  0.052186273  0.007613436  0.002783064  0.00338048  0.001833997  0.002930413  0.007694922  0.003049358  0.002113071  0.004095243 |
| --- | --- | --- | --- | --- | --- | --- | --- |

| rs9273363 | 0.020868381 | A | C | 0.645014472 | 0.279183862 | 990.541894 | 0.014866184 |
| --- | --- | --- | --- | --- | --- | --- | --- |
| rs941823 | 0.633117514 | C | T | -0.313186906 | 0.656103795 | 169.6726022 | 0.002578232 |
| rs9457247 | 0.72101426 | T | C | -0.194526141 | 0.544733093 | 260.2248847 | 0.003948771 |
| rs9557207 | 0.293512926 | G | A | -0.862576954 | 0.82115276 | 176.0591817 | 0.002675019 |
| rs974801 | 0.743916904 | G | A | 0.223396975 | 0.683859682 | 164.1877638 | 0.002495096 |
| rs9836291 | 0.242333821 | A | G | -0.359682844 | 0.307638608 | 703.8127118 | 0.010608566 |
| rs9889296 | 0.739260105 | A | G | 0.162504405 | 0.488245602 | 288.0517911 | 0.004369184 |
