# Supplementary figures and images for: Investigating causal associations between inflammatory bowel disease and IgA vasculitis: Univariable and multivariable Mendelian randomization study
Source: Medicine (Baltimore). 2026 Jul 24;105(30):e49953. doi: 10.1097/MD.0000000000049953 (PMC13406238; doi:10.1097/MD.0000000000049953)

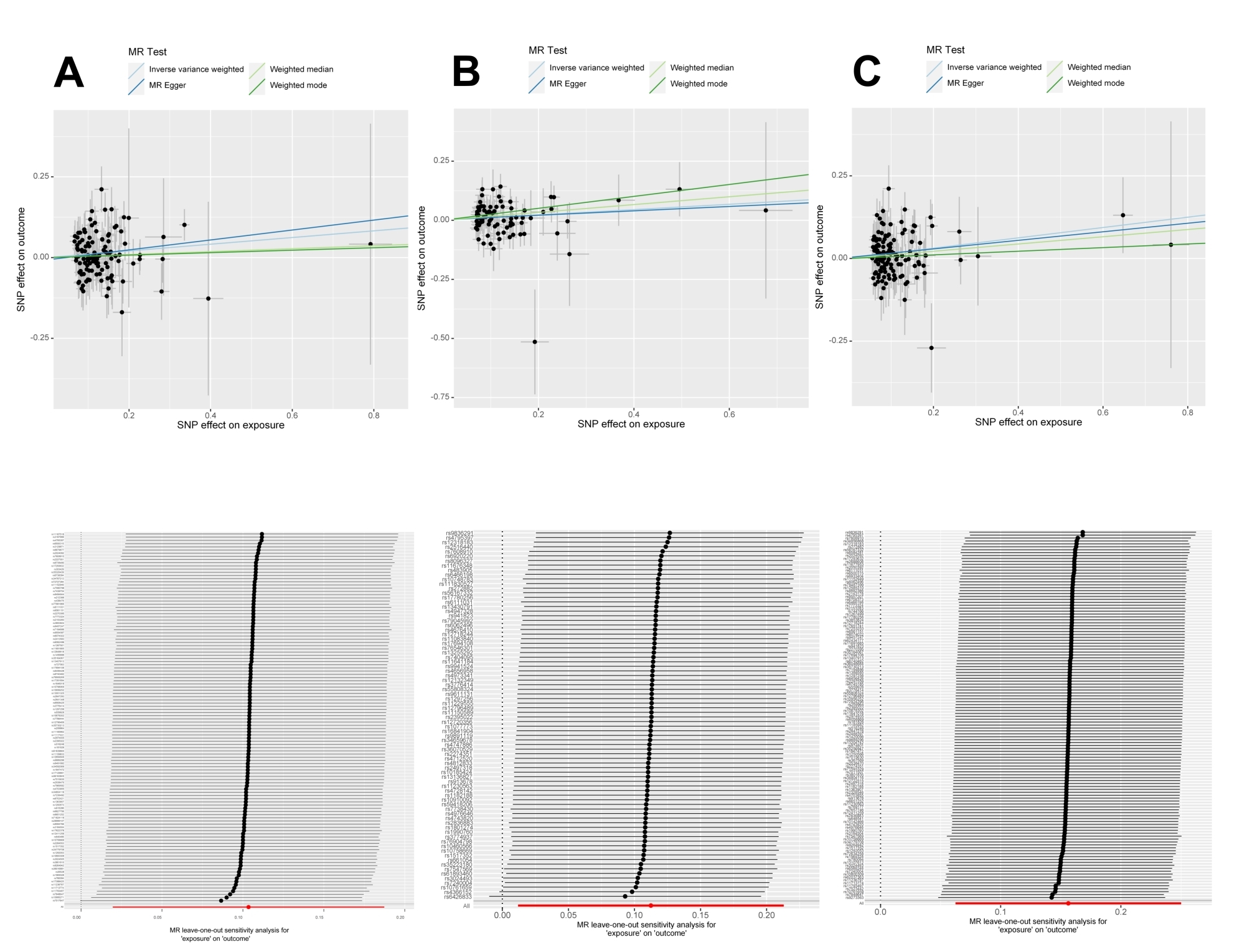

Supplement: Supplementary file 2 [file medi-105-e49953-s002.jpg]

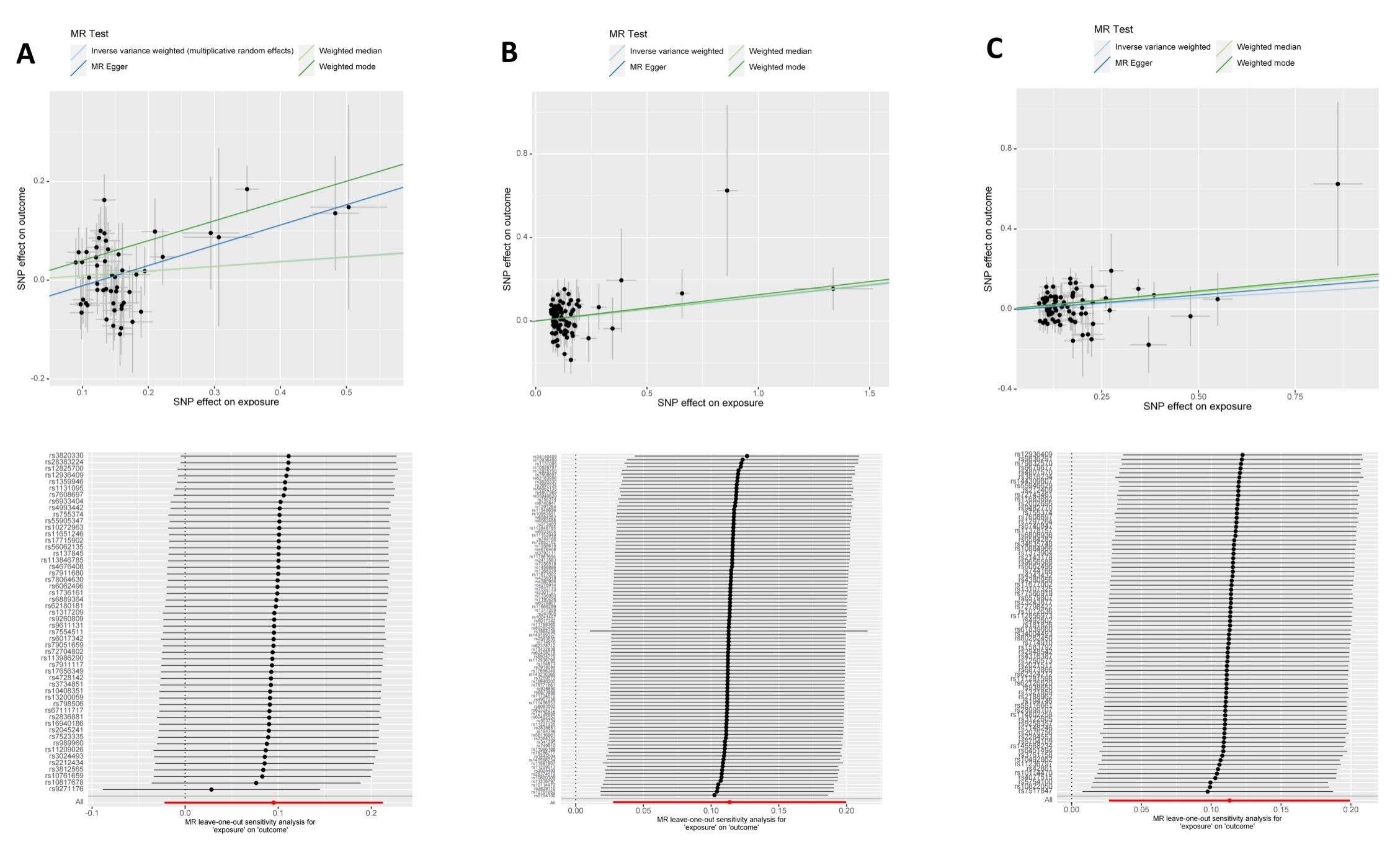

Supplement: Supplementary file 3 [file medi-105-e49953-s003.jpg]
