# Supplementary material for: Investigating causal associations between inflammatory bowel disease and IgA vasculitis: Univariable and multivariable Mendelian randomization study
Source: Medicine (Baltimore). 2026 Jul 24;105(30):e49953. doi: 10.1097/MD.0000000000049953 (PMC13406238; doi:10.1097/MD.0000000000049953)
Supplement: Supplementary file 4 [file medi-105-e49953-s004.doc]

**Table S2. MR and Sensitivity analysis of IBD,UC and CD(IIBDGC database) with the risk of IgAV**

**Supplementary Table S2. MR and Sensitivity analysis of CD with the risk of IgAV.**

| Method | OR 95% CI | P value |
| --- | --- | --- |
| IVW | 1.11 1.02- 1.21 | 0.0156 |
| Weighted median method  MR-Egger regression | 1.05 0.92- 1.19  1.17 0.93- 1.47 | 0.4805  0.1946 |
| MR-PRESSO  methoda  Heterogeneityb  Pleiotropyc | na  I2 = 9.4% ; Cochrane's Q =127; Intercept = -0.007; Pple = | Phet = 0.21 0.65 |

a na means there is no outlier needed to be corrected. b No significant heterogeneity was observed in the analysis. c MR-Egger was used to detect Pleiotropy.

**Abbreviation:** CD, Crohn's disease. IgAV, IgA vasculitis.

**Supplementary Table S2. MR and Sensitivity analysis of UC with the risk of IgAV.**

| Method | OR | 95% CI | P value |
| --- | --- | --- | --- |
| IVW | 1.12 | 1.01- 1.24 | 0.0284 |
| Weighted median method | 1.18 | 1.01- 1.37 | 0.0326 |
| MR-Egger regression | 1.10 | 0.85- 1.41 | 0.4737 |
| MR-PRESSO  methoda |  | na |  |
| Heterogeneityb | I^2^ = | 1.6% ; Cochrane's Q | = 80; Phet = 0.44 |
| Pleiotropyc |  | Intercept =0.003; | Pple = 0.87 |

^a^ na means there is no outlier needed to be corrected. ^b^ No significant heterogeneity was observed in the analysis. ^c^ MR-Egger was used to detect Pleiotropy.

**Abbreviation:** UC Ulcerative colitis, IgAV, IgA vasculitis

**Supplementary Table S2. MR and Sensitivity analysis of IBD with the risk of IgAV.**

| Method | OR | 95% CI | P value |
| --- | --- | --- | --- |
| IVW | 1.17 | 1.06- 1.28 | 0.0011 |
| Weighted median method | 1.12 | 0.97- 1.28 | 0.1252 |
| MR-Egger regression | 1.14 | 0.91- 1.43 | 0.2672 |
| MR-PRESSO  methoda |  | na |  |
| Heterogeneityb | I^2^ = | 6.3% ; Cochrane's Q = 133; | Phet = 0.29 |
| Pleiotropyc |  | Intercept = 0.003; Pple = | 0.80 |

^a^ na means there is no outlier needed to be corrected. ^b^ No significant heterogeneity was observed in the analysis. ^c^ MR-Egger was used to detect Pleiotropy.

**Abbreviation:** IBD inflammation bowel disease, IgAV, IgA vasculitis
