# Supplementary material for: Investigating causal associations between inflammatory bowel disease and IgA vasculitis: Univariable and multivariable Mendelian randomization study
Source: Medicine (Baltimore). 2026 Jul 24;105(30):e49953. doi: 10.1097/MD.0000000000049953 (PMC13406238; doi:10.1097/MD.0000000000049953)
Supplement: Supplementary file 5 [file medi-105-e49953-s005.doc]

**Table S3. MR and Sensitivity analysis of IBD,UC and CD (de Lange et.al database) with the risk of IgAV**

**Supplementary Table S3. MR and Sensitivity analysis of CD with the risk of IgAV.**

| Method | OR | 95% CI P value |
| --- | --- | --- |
| IVW | 1.12 | 1.03- 1.22 0.0102 |
| Weighted median method | 1.18 | 1.05- 1.34 0.0068 |
| MR-Egger regression | 1.17 | 0.93- 1.47 0.1907 |
| MR-PRESSO  methoda |  | na |
| Heterogeneityb | I^2^ = | 17.7% ; Cochrane's Q =91; Phet = 0.099 |
| Pleiotropyc |  | Intercept = -0.008; Pple = 0.70 |

a na means there is no outlier needed to be corrected. b No significant heterogeneity was observed in the analysis. c MR-Egger was used to detect Pleiotropy.

**Abbreviation:** CD, Crohn's disease ，IgAV, IgA vasculitis

**Supplementary Table S3. MR and Sensitivity analysis of UC with the risk of IgAV.**

| Method | OR | 95% CI P value |
| --- | --- | --- |
| IVW | 1.10 | 0.98- 1.24 0.1119 |
| Weighted median method | 1.10 | 0.93- 1.29 0.2683 |
| MR-Egger regression | 1.51 | 1.08-2.10 0.0189 |
| MR-PRESSO  methoda |  | rs9271176 |
| Heterogeneityb |  | I2 = 29.9% ; Cochrane's Q = 73; Phet = 0.02 |
| Pleiotropyc |  | Intercept =-0.052; Pple = 0.05 |

a No significant heterogeneity was observed in the analysis. bMR-Egger was used to detect Pleiotropy.

**Abbreviation:** UC, Ulcerative colitis, IgAV, IgA vasculitis

**Supplementary Table S3. MR and Sensitivity analysis of IBD with the risk of IgAV.**

| Method | OR | 95% CI P value |
| --- | --- | --- |
| IVW | 1.12 | 1.03- 1.22 0.0096 |
| Weighted median method | 1.12 | 0.96- 1.31 0.1334 |
| MR-Egger regression | 1.12 | 0.97- 1.30 0.1241 |
| MR-PRESSO  methoda |  | na |
| Heterogeneityb |  | I2 = 13.7% ; Cochrane's Q = 110; Phet = 0.14 |
| Pleiotropyc |  | Intercept =-0.0005; Pple = 0.96 |

a na means there is no outlier needed to be corrected. b No significant heterogeneity was observed in the analysis.

c MR- Egger was used to detect Pleiotropy.

**Abbreviation:** IBD, inflammation bowel disease ，IgAV, IgA vasculitis
